# Integrating Prognostic Breeding Approach Through Phenotypic and Marker-Assisted Selection for Yield and BCMV Resistance in Common Bean Greek Landraces

**DOI:** 10.3390/plants15060963

**Published:** 2026-03-20

**Authors:** Eirini N. Demertzi, Lefkothea Karapetsi, Chrysanthi I. Pankou, Nefeli Vasileiou, Eleftheria Georgiadou, Anastasia Kargiotidou, Varvara I. Maliogka, Dimitrios Vlachostergios, Panagiotis Madesis, Athanasios G. Mavromatis

**Affiliations:** 1 Laboratory of Genetics and Plant Breeding, School of Agriculture, Aristotle University of Thessaloniki, 54124 Thessaloniki, Greece; edemertzi@agro.auth.gr (E.N.D.); riogeo01@gmail.com (E.G.); 2 Institute of Applied Biosciences (INAB), Centre for Research & Technology Hellas (CERTH), 57001 Thessaloniki, Greece; lefki8@certh.gr (L.K.); pmadesis@certh.gr (P.M.); 3 Laboratory of Plant Molecular Biology, Crop Production & Rural Environment, Department of Agriculture, University of Thessaly, 38446 Volos, Greece; 4 Institute of Industrial & Forage Plants, Hellenic Agricultural Organization “DIMITRA”, 41335 Larissa, Greece; kargiotidou@elgo.gr (A.K.); vlachostergios@elgo.gr (D.V.); 5 Laboratory of Plant Pathology, School of Agriculture, Aristotle University of Thessaloniki, 54124 Thessaloniki, Greece; vmaliogk@agro.auth.gr (V.I.M.)

**Keywords:** genetic diversity, landraces, low-input agriculture, SSR, SCAR, CAPS molecular markers, *Phaseolus vulgaris* L., sustainability

## Abstract

Addressing principal challenges in common bean (*Phaseolus vulgaris* L.) breeding requires a holistic approach. A combined strategy was implemented to assess seven genotypes (landraces and commercial varieties) for yield potential, stability and resistance to bean common mosaic virus (BCMV) under Mediterranean low-input conditions. Pure-line selection and prognostic breeding together with SSR and CAPS-SCAR marker-assisted selection (MAS) formed the core methodology. Significant variation was detected across 24 morpho-agronomic descriptors, while SSR revealed 48.57% polymorphic loci and private alleles in specific landraces. High genetic coefficients of variation and high heritability were recorded for yield-related traits. Phenotypical evaluation showed diverse responses to BCMV, with mild symptoms predominating (52.14%). Entries G1 (45%) and G5 (35%) exhibited the highest frequency of the symptomless resistant phenotype. Molecular screening at *I* and *bc-3/eIF4E* loci confirmed G5’s robust dominant *I* gene profile, while G1 included individuals carrying both the dominant *I* gene and recessive *bc-3*, offering a valuable source for pyramiding resistance. Additionally, G1 (LI = 2.35; 100%) performed strongly in productivity, whereas G2 (SI = 3.1; 100%) and G7 (SI = 2.8; 89.7%) exhibited exceptional stability. Overall, the mixed-model approach highlighted the complementary characteristics of the tested genotypes and identified G1, G2, G5 and G7 as promising candidates for future breeding programs targeting high yield, low-input adaptability and resistance to BCMV.

## 1. Introduction

Common bean (*Phaseolus vulgaris* L.) is a major grain legume of the *Fabaceae* Lindl. family underpinning global food and nutritional security, serving as both a staple protein source and a fresh vegetable. Dry beans account for a high percentage of daily protein intake in many countries, particularly in Latin America, Africa, and Asia. According to the most recent FAOSTAT statistics, cultivation covered approximately 33.7 million hectares globally in 2024, yielding 30.3 million tons with an average productivity of 898 kg/ha (FAOSTAT, 2024) [1]. In Greece, the most recent available data from 2017 indicate a cultivated area of 8030 hectares, producing 18,013 tons with an average yield of 2243 kg/ha. This superior productivity (~2.5-fold higher than the current global average) reflects the favorable agroecological conditions of Greek bean-growing regions (Macedonia, Thrace and Thessaly), while highlighting the crop’s significant contribution to national agricultural production. In certain cropping areas, farmers have sustained the practice of cultivating specific common bean landraces. Nevertheless, the majority of traditional cultivars have undergone a progressive replacement with elite commercial varieties, to facilitate higher yields and consequently higher income, thereby aligning with the requirements of both farmers and consumers [2].

However, the future integrity of the crop is threatened by yield instability induced by multiple abiotic and biotic factors, across different cultivation systems and environments. Particularly, the susceptibility to viral diseases is a critical inhibiting factor, resulting in substantial economic losses. Furthermore, the narrowing of the genetic base resulting from domestication has probably further reduced the crop’s adaptive capacity to certain stresses, such as drought conditions and specific pathogens [3]. Bean common mosaic virus (BCMV), a member of the genus *Potyvirus*, is the most frequent virus affecting common bean worldwide [4]. This non-persistent aphid-transmitted virus, which can also be seed-transmitted with up to an 80% efficiency, causes mosaic, chlorosis, etiolation, and deformation, plant retardation, local or systematic necrosis, and even whole-plant necro-sis (WPN) with specific strains [5].

Addressing this virus requires the identification and utilization of resistant genotypes through germplasm screening and incorporation into breeding programs. Resistance towards BCMV is governed by a single dominant (*I*) gene and a few recessive genes (*bc-1*, *bc-2*, *bc-3*, *bc-4*, *bc-u^d^*, and *bc-u^r^*) [6]. Genotypes carrying the dominant (*I*) gene possess resistance against BCMV through a hypersensitive response (HR) on the primary leaves, while recessive genes confer absolute resistance toward specific viral strains [6]. Necrotic BCMV strains (NL-2 and NL-6) require high temperatures (>30 °C) to trigger WPN in genotypes bearing the *I* gene, necessitating the combination of the “*I*” gene with extra “*bc*” recessive genes, especially in high-temperature areas [7]. However, even in the case of non-necrotic strains, gene pyramiding with recessive genes will further protect the host and contribute to durable resistance. With the exception of BCMV isolate 1755a (PG-VIII), which managed to overcome *bc-3* resistance, the *bc-3* gene is remarkable as it confers resistance to nearly all BCMV strains [8].

According to the principles of the European Green Deal, there is a great need for the characterization and conservation of germplasm resources contributing to food security, as advocated by the 2030 Agenda. Considering the dynamic contribution of legumes—especially common bean—in addressing the climate crisis consequences, prior knowledge of the genetic variability in each region is a prerequisite for the development of more productive and resilient varieties [9]. This need is intensified by the reduction in cultivated areas and the lack of registered varieties with adaptability to local conditions. Local bean landraces represent valuable genetic resources, offering beneficial variability that provides them with high adaptability, resistance to biotic and tolerance to abiotic stress and occasionally the presence of specific quality traits. Hence, they constitute a valuable gene pool for the accumulation of favorable genes in order to broaden the genetic base and, at the same time, to maximize the gains from selection and obtain superior pure lines adapted to target environments. For all these reasons, their conservation and characterization are essential for further improvement of productivity and quality of the final product under sustainable conditions [10]. Additionally, adequate variability provides options from which selections for possible hybridization may arise [11].

Field screening for biotic challenges still needs standardization and improved methodologies. Furthermore, a breeding approach that could differentiate resistant from susceptible single plants at the early stages of selection is a prerequisite for the development of resistant varieties in order to avoid the loss of valuable genetic variability that exists in early generations [12]. Honeycomb breeding is suggested as an innovative phenotyping method for developing improved varieties under minimal interplant competition, as it maximizes phenotypic differentiation and eliminates the masking effects induced by the negative relationship between yield and competitive ability [13,14,15,16]. Particularly under stress conditions, resistance or susceptibility is often more clearly expressed when plants are widely spaced, facilitating the identification of superior genotypes [17,18,19,20].

At the molecular level, the use of two complementary marker systems is indispensable for continuous marker-assisted screening in breeding pipelines [21]. Marker-assisted selection (MAS), along with field-phenotyping, serves as a means to efficiently select genotypes with desirable resistance alleles in early generations, accelerating the process. Cost-effective Simple Sequence Repeat (SSR) markers remain valuable for evaluating genetic variability, population structure, and allelic richness in common bean germplasm [22]. Assessing genetic background remains critical for choosing parents and for anticipating genotype × environment responses, while such diversity analyses can be interpreted along with agronomic performance and MAS to position distinct donors that also satisfy yield and resistance criteria. In parallel, molecular markers linked to virus-resistant genes provide rapid and reliable identification of genotypes carrying key alleles against BCMV. The dominant *I* gene, conferring resistance to most BCMV pathotypes, is routinely monitored through the SCAR marker SW13, derived from a tightly linked RAPD fragment [23]. The *I* locus is also detected by codominant and tightly linked CAPS markers, which were developed through in silico bulked segregant analysis (BSA), controlling resistance to BCMV [24]. The recessive *bc-3* locus, corresponding to an allele of the eukaryotic translation initiation factor *eIF4E*, is widely used to detect specific polymorphisms after restriction enzyme digestion [25,26].

Recent advances in common bean breeding have increasingly emphasized the integration of phenotypic and molecular approaches across multiple breeding objectives. For genetic diversity assessment, Kumari et al. [27] identified elite donors via agro-morphological characterization and anthracnose disease screening using a 1–9 scale under natural epiphytotic conditions, while Raja et al. [28] aimed to uncover the genetic distinctions among common bean genotypes from the Nilgiri Hills through morphological, biochemical, and SSR markers. Regarding yield improvement, Reinprecht et al. [29] sought to identify regions in the common bean genome associated with yield and a number of yield-related traits using a collection of 121 diverse bean genotypes with different yields genotyped with SNP markers. In addition, Shivanchi et al. [30] attempted to pyramid multiple-disease resistance in common bean breeding lines based on data recorded on disease severity and agronomic traits. For BCMV resistance, Smail et al. [31] also detected differentiation between resistant and susceptible genotypes based mainly on visual symptoms, while Soler-Garzòn et al. [32] aimed to identify and validate structural variations for the *bc-u^d^* gene, develop an efficient DNA marker to assist selection of *bc-u^d^* in dry beans, and examine the interactions between the *bc-u^d^* allele and other BCMV resistance genes. However, to our knowledge, no previous study has integrated the honeycomb prognostic breeding methodology with SSR diversity analysis and BCMV resistance markers for simultaneous selection of yield potential, stability, and disease resistance in common bean.

In this context, we applied a combined phenotypic and molecular screening methodology that included single-plant selection at ultra-low density under low-input conditions by quantifying agronomic performance and stability, using SSR genotyping to map the diversity of landrace germplasms, and implementing targeted MAS to find common bean genotypes carrying BCMV resistance alleles. The primary objective of the project was to detect the ideotype, which reflects adaptability to low-input sustainable environments and disease pressure, for integrating into future breeding programs. The implementation of the proposed dual approach could (i) enhance early-generation selection by harmonizing yield performance with stability, (ii) identify elite genotypes and advanced lines possessing advantageous resistance genes, and (iii) reveal genetically distinct donors/parents for crosses—yielding practical, field-validated results consistent with the fundamental principles of common bean breeding.

## 2. Results and Discussion

### 2.1. Genetic Diversity Using Morphological and Molecular Approaches

We investigated the diversity of common bean resources widely grown in Greece, based on 24 morphological and agronomic descriptors but also five SSR markers, for incorporation into subsequent breeding programs. Primary visual observation confirmed the predominance of a distinct market class. Among the 12 qualitative descriptors, 6 were polymorphic, with terminal leaflet shape showing the highest diversity (6 categories). The relatively low polymorphism in qualitative traits (50% monomorphic) compared to quantitative reflects strong directional selection for preferred phenotypes. Especially for the flower color, 100% of genotypes presented a white standard and wings. This observation is in accordance with the results from other studies, where the white character predominated [9,33,34]. Regarding the seed color, 100% of genotypes had white seed coats. The white seed color in common bean results from convergent evolution in the *P* (pigment) gene [35,36]. The recessive *p* allele is pleiotropic to other genes in the network, and *pp* homozygotes produce white seeds together with white flowers [37]. The prevalent white phenotype unravels the allelic distribution of the tested germplasm. However, the *P* gene has also been used as the first genetic marker for a quantitative trait because a QTL for seed size is linked to it. The association with seed size indicates that maybe the predominance of the white phenotype implies a continuous selection preference for specific ecotypes. In a similar study by Nadeem et al. [34], the most dominant seed color was also white.

Regarding the plant type, 71.43% of genotypes were characterized as indeterminate type II growth habit, according to CIAT guidelines [38]. According to Kläsener et al. [39], indeterminate growth habit contributes to better grain quality since pods do not touch the soil, while Nasar et al. [40] described it as an ecophysiological adaptation to increase light exposure and photosynthetic efficiency. For the terminal leaflet shape, 77.14% were circular to rhombic, followed by rhombic (14.29%), triangular (2.86%), triangular to circular (2.86%), and circular (2.86%) leaflets. For the pod shape in the cross section, two categories were reported among genotypes (ovate 74.29% and elliptic 25.71%). At the same time, 48.57% of the seeds were kidney-shaped in the longitudinal section, 22.86% were rectangular, 20% were circular to elliptic, and 8.57% were elliptic, indicating a strong acceptance of kidney-shaped grains among European (especially Mediterranean) consumers. A similar study by Jan et al. [33] reported kidney (26.6%), cuboidal (28.4%), and circular to elliptical shapes (28.4%) as the most predominant. It is interesting to note that a correlation was identified between specific qualitative traits, e.g., plant type and seed shape, plant type and pod shape of the distal part, pod shape of the distal part and seed shape, and pod shape in the cross section and pod stringiness of the ventral suture, enabling the concurrent and indirect selection for these descriptors, in consistency with previous studies [41]. Characterization based on such qualitative traits enables separation of the genotypes into groups representing different geographical areas or different races of the same gene pool [42]. As reported by Dutta et al. [43] and Zeven et al. [44], such morphological markers often co-vary and contribute to germplasm differentiation.

For the quantitative traits, a statistically significant difference was found among the entries for 11 out of 12 evaluated descriptors (Table 1), indicating variability between the tested genotypes. The only trait for which no statistical significance was detected was pod width, suggesting a strong environmental influence. It is extremely noteworthy that the average 100-seed weight observed was 50.89 g, consistent with previous studies that reported similar values for Greek landraces of indeterminate type under normal irrigation and relatively low plant density [45]. In the study by Rana et al. [46], the average value was 27.5 g, while De Paula et al. [9] reported an average value of 21.43 g. Similarly, Mesera et al. [47] observed a mean value of 17.90 g. Our findings showed higher values compared to the above-mentioned studies, which reported average values for 100-seed weight that ranged from 17.9 g to 27.5 g [9,46,47]. This difference can be attributed to several factors. First, the honeycomb prognostic breeding design employed in our study eliminates intergenotypic competition through systematic plant spacing, allowing for maximum phenotypic differentiation and full expression of genetic potential [19], particularly for yield-related traits. Second, growth habit differences substantially influence seed size and yield components in common bean, with indeterminate types II, III, and IV showing advantages over determinate types [48]. Additionally, field evaluation conditions versus controlled greenhouse experiments can result in different trait expression patterns due to resource availability and genotype × environment interaction. In a related study by Kargiotidou et al. [49] involving dense sowing, a 100-seed weight equal to 36.25 g was reported specifically for G1 (Pyrgetos), while for G4 (Lingot), the same descriptor was equal to 29.25 g. Our findings are very close to those obtained in a previous study by Mavromatis et al. [10], in which the evaluated Greek landraces of indeterminate growth habit (IV) showed similar values for 100-seed weight (28.67–59.82 g). This observation—by providing indirect information about the seed size—suggests the emblematic Mediterranean “local bean”. It is also imperative to acknowledge the mean agronomic performance of the evaluated germplasm for the number of pods and number of seeds per plant. The number of pods per plant observed in the current study was 58.40 and the number of seeds per plant was 153.59. Papathanasiou et al. [45] reported an average number of pods per plant equal to 91.54 when evaluating climbing (indeterminate growth habit IV) landraces adapted to the Mediterranean climate. This advantageous characteristic indicates the high yield potential of local (Greek) landraces and their possible utilization in future breeding programs to improve the seed yield of common bean. Additionally, correlation analysis revealed significant pairwise correlations among quantitative traits (*p* < 0.05). A strong negative correlation between the number of pods per plant and 100-seed weight (r = −0.79; *p* < 0.05) suggests either a pleotropic effect or tight genetic linkage and has important breeding implications through indirect selection for one trait or the other. This negative correlation between seed size and yield should be considered carefully when selecting the best-performing genotype, since the preference for large-seeded cultivars by consumers probably derives from assumptions of their rapid hydration and satisfactory taste [50].

After the analysis of variance, the means of the morphological and agronomical data were subjected to Skott–Knott mean clustering (*p* < 0.01; Table 1). The descriptor seed length presented the largest number of classes—four in total—with values ranging from 7.80 mm (G6—Karatzova Landrace) to 13.60 mm (G4—Lingot). The plant height, leaflet width, pod length of beak and seed width each comprised three groups. For the plant height, the values ranged from 48.50 cm (G4—Lingot) to 121 cm (G1—Pyrgetos), with an average value of 83.31 cm. The statistically significant positive correlation between plant height and number of pods per plant observed in our study suggests a possible correlation between plant type and yield components, confirming the advantage of indeterminate growth habit genotypes [48]. Especially for the Greek landraces, the distribution of pods in the upper half of the plant also favors the harvestability, resulting in special breeding contributions.

The present study found that GCV (%) values varied from 8.39% for pod length to 38.05% for plant height. Characters with a high GCV value (>20%) were plant height (38.05), pod length of beak (29.76), number of pods per plant (26.19), number of seeds per pod (29.28) and 100-seed weight (24.46), according to the categorization of Burton et al. [51]. The elevated GCV estimations for these traits indicate the limited expression of environmental variation across the tested genotypes and suggest that yield increase through phenotypical selection is feasible. These results are consistent with Wondimu et al. [52] and Kuma et al. [53], who revealed high GCV values for the number of pods per plant, number of seeds per pod and 100-seed weight. Estimated values for broad-sense heritability (*H*^2^) for the 12 quantitative traits ranged from 29% for pod length and thickness to 86% for plant height. In particular, plant height, leaflet width, pod length of beak, seed length and seed width exhibited high heritability (>60%), according to the categorization of Johnson et al. [54]. Respectively, leaflet length, the number of pods per plant, number of seeds per pod and 100-seed weight demonstrated medium heritability values (30–60%). Kargiotidou et al. [49] also reported high heritability for 100-seed weight (79%). Strong heritability estimates for these traits indicates that the variation observed was mainly under genetic control and was less influenced by the environment, implying that selection for these characters would be effective due to additive gene action. The relatively low standard errors of broad-sense heritability (*H*^2^) for specific traits indicate good precision of the genetic parameter estimates. The combined elevated values of GCV% and *H*^2^ for the above morpho-agronomic traits indicate the high potential for genetic advancement for these characters. Furthermore, high heritability estimates along with a high genetic advance as a percentage of mean (GAM > 20%) were found for traits such as plant height, leaflet width, pod length of beak, seed length and seed width, making the direct selection effective for the improvement of these traits. The combination of the two genetic parameters typically improves the prediction of genetic gain under selection [54].

**Figure 1:**
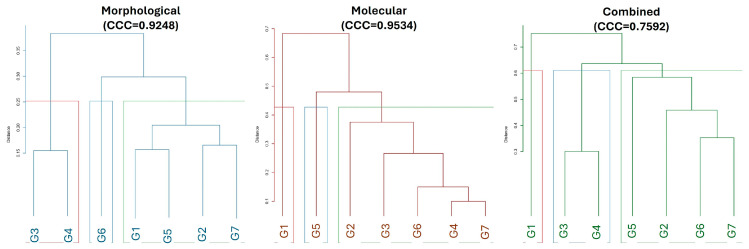
UPGMA dendrograms based on morphological, molecular and combined matrices for the 7 evaluated common bean entries. Optimal number of clusters *k* = 2, according to Silhouette analysis. Note: CCC > 0.90 excellent fit; CCC 0.80–0.90 good fit; CCC 0.70–0.80 acceptable fit. Created with R software version 4.2.2.

The dissimilarity matrix, through the Gower algorithm (incorporating both quantitative and qualitative traits), showed that based on the morpho-agronomic descriptors, the evaluated genotypes have an average genetic distance of 0.30 (±0.10), ranging from 0.15 (G3–G4) to 0.44 (G3–G5). The medium average distance coupled with the higher distances noted between landraces and reference genotypes (commercial varieties Lingot and Cannellino) highlighted the uniqueness of this material. Therefore, relative homogeneity was observed among the tested genotypes—though not restrictive—accompanied by increased intrapopulation variability, promoting the selection of promising lines. The dissimilarity found is notable for the recombination of parental characteristics and the effective selection of superior individuals for segregating generations [55]. UPGMA clustering based on Gower dissimilarity produced a dendrogram with a cophenetic correlation coefficient (CCC) of 0.92, indicating excellent fit (Figure 1). The high CCC value (>0.90) confirms the reliability of the grouping [56].

The dendrogram grouped genotypes into three major clusters. Cluster I (reference genotypes G3–G4) was characterized by erect-growing plants with a lower plant height, short internode length, decreased node number (3–5) and apical growth terminating in an inflorescence (determinate type I). These two commercial varieties were also characterized by a decreased number of pods per plant and number of seeds per pod, a greater seed size and early flowering. Cluster III (G1, G2, G5, and G7) exhibited contrasting features related to plant type (indeterminate type II) and yield parameters. These genotypes were characterized as upright short vines with few branches and pods, concentrated primarily in the middle of the plant, that demonstrated later flowering and longer season maturity. This observation further confirms the assumption that landraces have probably retained adaptation characteristics from the wild type. Entry G6 (landrace Karatzova) emerged as the most divergent, occupying a distinct cluster (Cluster II). Thus, this entry represents a useful genetic resource for expanding the genetic base through crossing programs because of its distinctive compact plant architecture and improved yield characteristics. This phenotypic differentiation likely reflects agronomic plasticity and adaptation to various agroecological zones.

**Figure 2:**
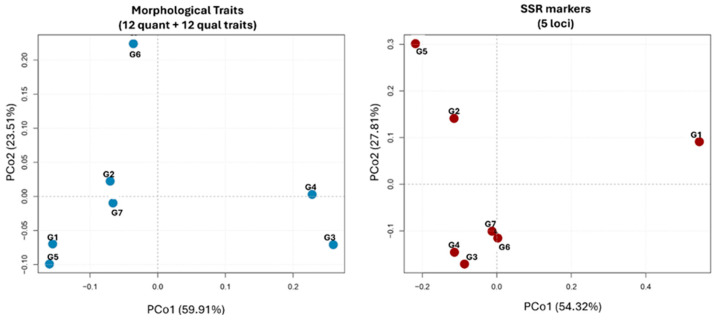
PCoA derived from morpho-agronomical and molecular matrices guiding the spatial distribution of the 7 common bean entries evaluated.

According to Silhouette analysis for morphological UPGMA, the optimal number of clusters was *k* = 2, validating the discrimination between Greek germplasm and control genotypes (G3 and G4). Hence, the clustering pattern based on Silhouette analysis partially aligns with geographical origin, market class and growth habit, suggesting the need for conservation. Principal coordinate analysis (PCoA) confirmed further the UPGMA results, with the first two coordinates explaining 59.91% and 23.51% (cumulatively 83.42%) (Figure 2). The ordination revealed that PCo1 primarily separated genotypes based on plant height, pod morphology and yield parameters, while PCo2 distinguished genotypes by leaflet characteristics, highlighting the direction that breeding should follow.

The five SSR loci amplified 19 alleles, with an average of 3.8 alleles per locus (range 139–258 bp). The average percentage of polymorphic loci was 48.57%, while private alleles were identified for SSR PhC-X04660 in landraces G2, G6, and G7 and for SSR PhC-AZ044945 (G6). Given that the two out of five SSR markers were monomorphic, the average values for Na (1.914), Ne (1.646), Shannon Index (0.426), He (0.252), and Ho (0.157) indicated moderate genetic diversity at neutral loci (Table 2). According to the Polymorphism Information Content (PIC), three out of five loci were classified as highly informative (PIC > 0.5). These specific markers could be particularly valuable for marker-assisted selection and genetic mapping in subsequent breeding programs.

Regarding the allelic patterns across the seven entries, the highest values for Na, Ne and the Shannon Index were estimated in landraces Karatzova (G6), Florina (G2) and Smyrni (G7), suggesting that this germplasm contains sufficient allelic variation for maintaining long-term genetic variability and responding to selection pressure. Additionally, the lowest Ho values observed in these entries point to the assumption that common bean landraces constitute mixtures of inbred lines, from which the direct selection of superior genotypes is effective. In summary, the moderate allelic richness despite the small sample size (*n* = 7) validates the fact that the studied genotypes capture substantial genetic variation, potentially representing distinct gene pools. UPGMA clustering based on Nei’s genetic distance revealed three clusters with a cophenetic correlation coefficient of 0.9534 (Figure 1), unraveling two distinct genotypes (G1 and G5), which could be used as potential promising parents in crossing schemes. Principal Coordinate Analysis (PCoA) for molecular data explained 82.13% of variance in the first two coordinates (Figure 2).

Concerning the combined analysis, a Mantel test revealed a very weak (not significant) correlation between morphological and molecular distance matrices (*r* = 0.0135; *p* = 0.474). This lack of correlation suggests that morphological traits are strongly influenced by environmental plasticity or that these specific loci do not directly control the corresponding traits. Previous studies have reported that several morphological traits of common bean, such as vine length and length of the main stem, depend strongly on environmental conditions (light, temperature, and moisture) [57]. Procrustes analysis yielded insignificant congruence between datasets, confirming the necessity of using both morphological and molecular markers to fully capture the available genetic variation. The combined approach integrating both morphological and molecular data (50:50 weight) produced a consensus dendrogram with CCC = 0.7592 (Figure 1), representing a balanced genetic structure incorporating both the type of variety (commercial vs. landraces) and origin (Greek germplasm vs. reference genotypes). Similarly, in other studies, no correlation was observed between the matrices of morpho-agronomic and molecular data [55].

### 2.2. Phenotypical Screening and Molecular Characterization for BCMV Resistance

Entry-specific resistance profile analysis revealed substantial variation in resistance responses according to the prevalent symptomatology (Table 3). Individual plants possessing mosaic and/or leaf rolling, systemic necrosis and intense weakness were classified as susceptible. On the contrary, plants showing hypersensitive necrotic reactions including top necrosis, vein necrosis and/or small necrotic lesions or mild mosaic were categorized as resistant/tolerant with mild symptoms, and finally plants with no symptoms were also characterized also as resistant. It is noteworthy that plants rated as resistant with no symptoms in the observation scale maintained this phenotype until the end of the biological cycle and were selected as promising genotypes. In the tested plants (20 per entry), BCMV was identified molecularly.

**Figure 3:**
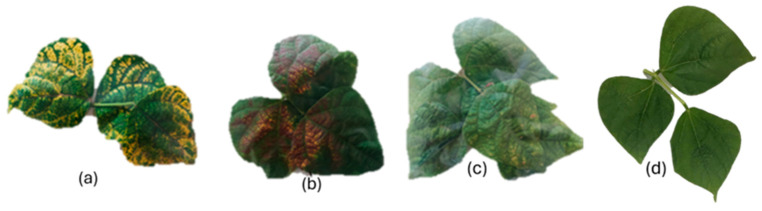
Visual symptoms caused by BCMV infection in common bean entries according to the type of symptoms: (**a**) mosaic and leaf rolling and (**b**) systemic necrosis: scale 1; (**c**) small necrotic lesions and mild mosaic: scale 2; (**d**) no symptoms: scale 3.

Phenotypic evaluation of BCMV resistance across the seven common bean entries revealed a predominantly resistant/tolerant germplasm, with 72.14% of plants exhibiting resistance responses. The overall phenotypical resistance distribution showed considerable variation, with 52.14% of plants classified as resistant/tolerant with mild symptoms (scale 2), 27.86% as susceptible with severe symptoms (scale 1) and 20% as resistant without visual symptoms (scale 3—absolute resistance phenotype) (Figure 3). This pattern indicated that mild symptoms represented the main response mechanism within the tested germplasm, while the symptomless phenotype, though less frequently observed, still existed. Our results are consistent with a previous study by Deligoz et al. [58], in which 75% of genotypes were recorded as resistant to BCMV after phenotypic analysis. The high proportion of the moderate resistance response (tolerance with mild symptoms) among the tested plants is commonly associated with the incomplete dominant nature of the *I* gene, as described by Collmer et al. [59], where *I*/*i* genotypes respond to BCMV infection through a hypersensitive response (HR). This resistance phenotype induced by mild necrosis of primary leaves was also demonstrated by Meghanath et al. [6]. The lower frequency of the symptomless resistance response suggests that this phenotype may be controlled by different resistance mechanisms or may require a specific genetic background for expression.

**Figure 4:**
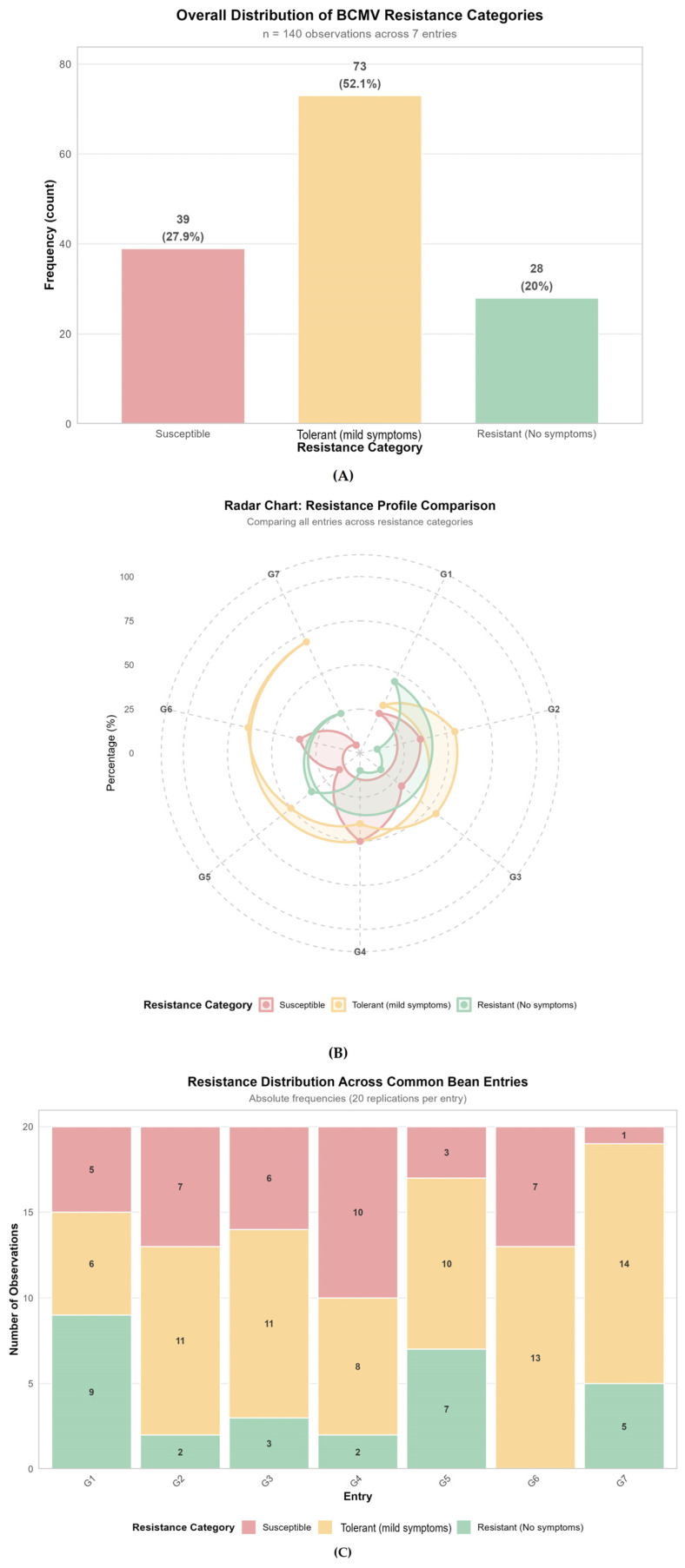
Entry-specific resistance profile analysis. (**A**) Overall distribution of BCMV resistance categories across the seven common bean entries; (**B**,**C**) BCMV resistance profile comparisons.

Based on the typical resistance category using the 20 observations per entry after phenotypical screening and virus molecular identification, six of the seven entries (85.7%) were classified as phenotypically resistant, with only G4 exhibiting a predominantly susceptible phenotype (Figure 4). Among the phenotypically resistant entries, G7 demonstrated the most robust resistance profile, with 70% of replications showing mild symptoms and only 5% severe symptoms. Similarly, G5 exhibited great resistance (85%), with 35% of tested plants possessing the symptomless phenotype. G2 and G3 showed intermediate resistance levels, with 55% of both entries presenting mild symptoms (tolerance to BCMV). Notably, G6 demonstrated strong tolerance with 65% displaying mild symptoms, while lacking the symptomless phenotype. Entry G1—known as an old Greek variety Pyrgetos—presented a more complex resistance profile, showing a relatively balanced distribution across all three categories (25% susceptible, 30% resistant/tolerant with mild symptoms and 45% resistant with no symptoms). This entry was the only one where the symptomless phenotype represented the most frequent response, suggesting potentially different underlying resistance mechanisms compared to other entries, e.g., the presence of recessive resistance genes or QTLs that confer resistance without visual symptoms. This observation defines the position of the Greek germplasm regarding the resistance profile. The superior resistance observed in the tested germplasm compared to commercial varieties (G3 and G4) underscores its value as a valuable gene pool for disease resistance. This finding aligns with numerous studies demonstrating that landraces represent important reservoirs of disease resistance genes that may have been lost during intensive commercial breeding focused on uniformity and yield [60]. This field-validated approach was followed as an alternative to typical practices in preliminary trials, in order to handle a large population size and combine it with the selection of superior genotypes under realistic conditions in the evaluation environment. Controlled trials should be performed subsequently to validate the reliability of the field data, in an attempt to adopt a systematic strategy for tracking genotypes for simultaneous selection in terms of yield and resistance.

Additionally, the chi-squared test of independence revealed a highly significant association between entry genotype and resistance phenotype (*X*^2^ = 28.666; *p*-value = 0.004416), indicating that resistance responses showed a genotype-specific pattern. This finding provides strong statistical evidence that genetic differences among entries influence their resistance to BCMV infection and different genotypes may carry distinct resistance genes or allelic variants that confer discrete phenotypes. This variation is extremely useful in breeding programs, as it indicates potential for pyramiding, in order to achieve durable resistance.

**Figure 5:**
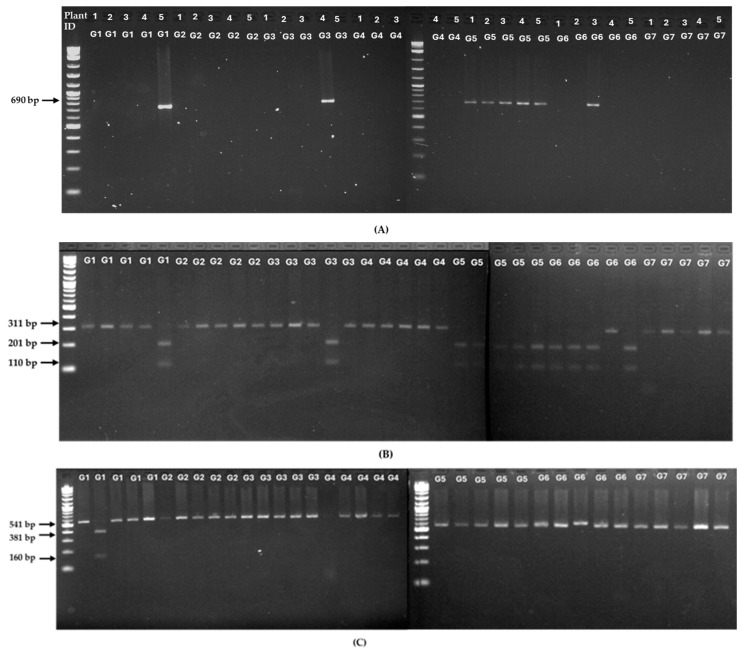
Amplification products for markers SW13 linked to the *I* gene (**A**), BCMV_CAPS linked to the *I* gene (**B**) and ENM-FWE/Rve linked to the *bc-3* gene. (**C**) Individual plants bearing the dominant *I* gene within the tested germplasm are labeled (**A**,**B**), while the recessive *bc-3* gene phenotype is also identified.

**Figure 6:**
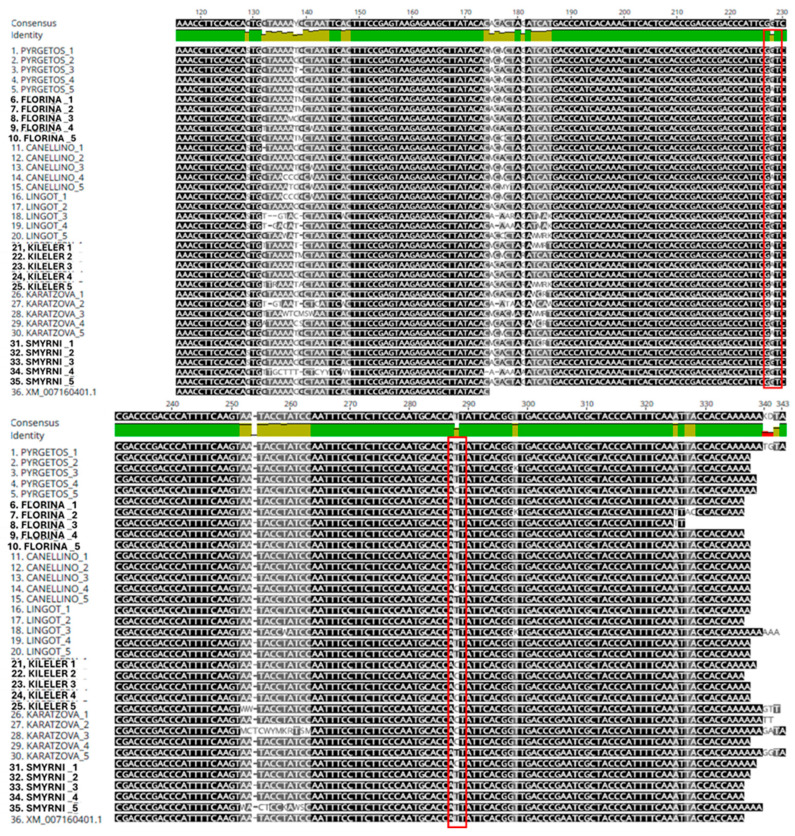
Sequence alignment of BCMV_CAPS PCR products, highlighting the point mutations of resistant genotypes. The Single-Nucleotide Polymorphisms (SNPs) identified in the tested germplasm are highlighted with red frames.

The observed phenotypic variation suggests the involvement of multiple resistance mechanisms operating within the tested germplasm. Indeed, the presence of the dominant *I* gene was confirmed initially by SCAR marker SW13 at the expected size of 690 bp, similarly to other studies [23,25,58] (Figure 5A). The BCMV-CAPS marker was later used for revalidation and interestingly, three more resistant genotypes were identified (Figure 5B) that had no SW13 marker-specific product. This erroneous genotyping is due to a recombination between the *I* gene and SW13-linked marker (linkage distance ~5 cM) [61]. PCR products of resistant genotypes (311 bp), upon digestion with TaqI (restriction site TCGA), generated products of 201 and 110 bp (Figure 5B), whereas susceptible plants remained un-cleaved due to a point mutation (TCGG). This Single-Nucleotide Polymorphism (SNP) was revealed after Sequence Alignment at 225–228 bp, identifying only truly resistant plants bearing the *I* gene (Figure 6). Another point mutation was identified at 288 bp (C/T), highlighting the discrimination between resistant and susceptible genotypes. The presence of the *bc-3* gene was confirmed by ENM-FWe/Rve marker. The digestion of PCR-amplified products (541 bp fragment) with RsaI cleaved a *bc-3* resistant genotype within entry G1 into 381 and 160 bp fragments, whereas susceptible genotypes remained un-cleaved, due to the absence of mutations within the *eIF4E* gene (Figure 5C). The existence of *bc-3* resistance emphasizes the possibility of achieving the *Ibc-3* combination through crossing schemes. A previous study reported that so far, the recessive *bc-3* gene has not been identified in Türkiye [58], boosting the value of Greek germplasm for disease breeding programs.

By combining phenotypic screening and amplification products for the plants presented in Figure 5, three potential resistance groups according to the genotype (allele combination) were assumed to shape the tested germplasm, expressed by two distinct phenotypes: no symptoms and mild symptoms (Table 4). The first group probably contains genotypes carrying the unprotected (without bc-3 recessive gene) dominant “*II*” gene, which were characterized by an absolute resistance response (no visual symptoms at all). This genotype had a higher frequency within entries G1 and G5 and was observed in all entries except for G6, which presented only the mild symptom response. In particular, G5 exhibited the highest frequency of resistance alleles across the tested plants, showing a robust dominant *I* gene profile. The second group is characterized by the unprotected “*Ii*” gene and presented mild symptoms during the phenotyping period. This genotype is assumed to be the most frequent across all entries. Finally, the third group is described as the *ibc-3* allelic combination, also presenting absolute resistance (no visual symptoms). Entry G1, which contains individuals bearing the dominant *I* gene or recessive *bc-3* gene, is a valuable source for pyramiding the desirable *Ibc-3* gene combination, through hybridizations within variety or intercross with G5. As expected, the unprotected ii allele combination was accompanied by severe symptoms in the majority of the cases tested. However, a noteworthy observation is the existence of phenotypically resistant/tolerant plants that did not carry any of the *I* and *bc-3* genes (G2 and G7). This finding implies the action of different resistance mechanisms, e.g., recessive resistance (*bc-u*, *bc-1*, and *bc-2*) or quantitative resistance, and this needs to be studied in the future.

Regarding the breeding implications, the tested materials can serve multiple roles in breeding strategies. The resistant/tolerant landraces can be directly incorporated as parental lines in crossing programs to introgress resistance genes into elite cultivars, broadening the narrow genetic base. For example, G7 and G6, with their exceptionally high frequency of tolerance responses, represent particularly promising donor parents. Combining the tolerance trait from these entries with the unique symptomless resistance phenotype from G1 and G5 could produce more durable resistance by engaging multiple defense pathways. Furthermore, controlled crosses among the resistant entries, particularly between those showing distinct resistance phenotypes, followed by inoculation with BCMV and gene expression analysis, would reveal extensively the mechanism of resistance inheritance between generations. The superior resistance observed in the evaluated germplasm emphasizes the critical importance of conserving these materials. Our results underscore the need for continued in situ conservation and ex situ germplasm collections to preserve this genetic diversity for future breeding efforts.

### 2.3. Prognostic Breeding for Yield Potential and Performance Stability

Yield losses due to biotic or abiotic stress are primarily attributed to reduced stability of crop yield potential. However, this is a trait with a distinct genetic component that can be successfully selected for at the single-plant level under nil-competition and integrated into high-yielding cultivars. The honeycomb methodology targets efficient selection for three gene categories: genes that control yield potential, genes that confer tolerance to abiotic and biotic stress, and genes that control cultivar responsiveness to inputs [12]. Fasoula et al. [62] reported the term “prognostic breeding”, introducing selection equations with prognostic power that objectively phenotype and evaluate individual plants in real field conditions in the absence of interplant competition and soil heterogeneity. The equations predict crop performance through the main concept of the coefficient of homeostasis or the Stability Index (SI), and their usefulness concerns both early generation selection and nonstop selection within finished cultivars for the incorporation of adaptive (genetic or epigenetic) responses of plants.

**Figure 7:**
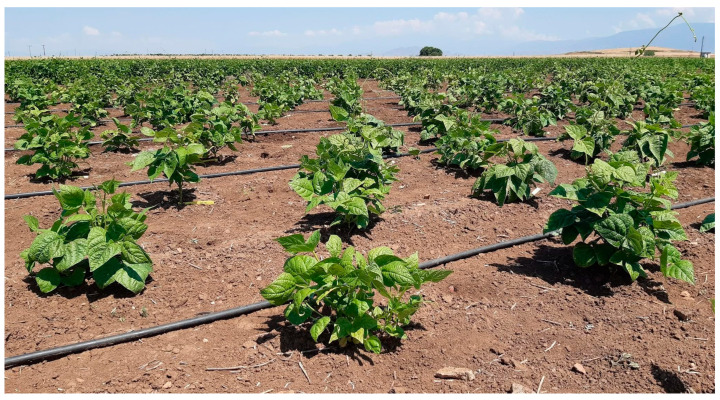
The honeycomb selection design R-7 is used to demonstrate the principles of moving complete replicates and grids and the components of the prognostic equations. Single plants of the same genotype formed triangular grids.

In the present study, honeycomb selection was implemented using a moving ring of 90 plants (Figure 7) to evaluate yield performance and stability across the seven common bean entries. Specifically for seed yield evaluation (g/plant), this systematic selection strategy employed two key prognostic equations: the Line prognostic equation (LPE = LI × SI) for ranking and selecting superior entries and Plant prognostic equation (PPE = PI × SI) for identifying elite plants within selected entries. Based on LPE values, entries were ranked to identify superior lines for advancement (Table 5).

Entry G1 (old Greek variety Pyrgetos) exhibited exceptional performance with the highest LPE value of 6.88 (100% relative performance), demonstrating both high yield potential (LI = 2.35; 100%) and excellent stability (SI = 2.9; 94.4%). This combination of high productivity and consistent performance across the low-input selection environment makes “Pyrgetos” the top-ranked entry. Landrace G2 “Florina” ranked second with an LPE value of 2.98 (43.2%), showing moderate yield potential but the highest stability index among the entries (SI = 3.1; 100%). Despite the lower yield performance, this exceptional stability indicates reliable performance across variable environmental conditions, a valuable trait for breeding programs targeting wide adaptability and plasticity in view of climate change effects. Landrace G7 “Smyrni” occupied the third position, demonstrating a balanced performance with an LI of 1.01 (42.9%) and SI of 2.8 (89.7%). The moderate-to-high performance across both yield and stability parameters, combined with its superior BCMV resistance profile, positions this landrace as a valuable genetic resource. Landrace G6 “Karatzova” ranked fourth, showing moderate yield potential but substantially lower stability, due to intrapopulation variability. Similarly, G5 “Kileler” ranked fifth, demonstrating both lower yield potential and reduced stability. Thus, these latter two entries may be used as donor parents for disease resistance alleles in crossing schemes with high-yielding entries, e.g., G1 or G2. The remaining entries, commercial varieties Lingot and Cannellino, ranked lowest, indicating limited yield potential but moderate stability of agronomic performance.

Regarding the PPE values, G1 again demonstrated excellent performance with Mean PPE value of 9.3 (100%), indicating the presence of numerous high-yielding individual plants. The standard deviation of 38 suggests considerable within-entry variation, allowing for continuous selection among individual plants. For landrace G2 (Florina), the second highest Mean PPE value (4.3; 46.41%) combined with exceptional stability suggests that the elite plants within G2 could serve as valuable parents in crossing programs, even though the overall performance of the entry was moderate. Additionally, landrace G7 “Smyrni” demonstrated adequate Mean PPE performance, making the selected plants from this entry particularly valuable germplasm. The remaining entries (G5, G3, and G4) showed lower Mean PPE values, indicating fewer elite plants for selection within the entries.

Considering a comparative analysis of resistance to BCMV and yield performance in the tested germplasm, a critical finding is that these two desired traits do not exclude each other, as evidenced by the superior performance of G1 across both evaluation systems. The combined analysis suggests a multi-trait selection strategy including targeted crossings between the tested materials, to increase the frequency of favorable alleles for both trait categories.

## 3. Materials and Methods

### 3.1. Plant Material and Experimental Design

The experiment was conducted during the 2023–2024 growing season at the Aristotle University Research Farm in Thessaloniki, Northern Greece (latitude 40°32′ S; longitude 22°59′ E; elevation 7 m a.s.l.). Seven common bean (*P. vulgaris* L.) entries were evaluated, comprising two European commercial varieties (Cannellino and Lingot) used as control genotypes, three landraces from different locations selected for their contrasting agronomic performances, one population of the Great Northern market type and one old Greek commercial variety. More specifically, “Pyrgetos” is an old Greek commercial variety (Landrace Karouba Lamias × Harvester) that has genetically diverged from its original type and exhibits variability due to long-term cultivation. Additionally, the two commercial varieties were chosen because, under Greek growing conditions, “Cannellino” seems to exhibit resistance to BCMV, while “Lingot” is known to be susceptible, effectively functioning as a positive and a negative check, respectively. The use of diversified genetic resources aimed to capture a wide representation of genetic and adaptive variability within the species. Seeds were visually inspected and standardized for size and vigor prior to sowing. The specific entries evaluated in this study are shown in Table 6.

The experimental design followed a honeycomb arrangement (R-7) [13] under nil-competition at the ultra-low density of 1.55 plants/m^2^, totaling 392 plants corresponding to 56 replicates per entry. The R-7 honeycomb selection design consisted of 14 rows, each containing 28 plants, with a spacing of 0.75 m between rows and 0.86 m between plants.

Prior to establishing the experiment, a soil analysis was conducted. The soil at the experimental site was classified as clay loam, with the following particle size composition: sand 29–59%, silt 25–40% and clay 27–34%. Furthermore, organic matter content was 1.4%, while the calcium carbonate equivalent (CaCO_3_) was recorded at 8%. Additionally, the electrical conductivity at 25 °C was low and total nitrogen (N) content was 0.12 g/100 g. Finally, the experimental field demonstrated a medium-low phosphorus content level. Micronutrient contents were Fe^2+^ 6.98 mg kg^−1^ and Zn^2+^ 0.42 mg kg^−1^, within adequate ranges for common bean cultivation. The pH was calculated to be 8.1 at 25 °C, indicating a slightly alkaline soil characterization. The soil preparation of the experimental plot involved primary tillage, followed by secondary tillage to achieve a fine seedbed. Cultivation practices and pest and disease control were performed following the recommendations for bean cultivation in low-input systems.

### 3.2. The Phenotypical Approach: Agro-Morphological Diversity, Virus Symptom Assessment and Prognostic Breeding for Superior Genotypes

A comprehensive phenotypic assessment was carried out to evaluate the morpho-agronomic diversity among entries and to estimate their response to bean common mosaic virus (BCMV), based on visual symptoms. A total of 24 morphological and agronomic traits were recorded, including 12 that follow a quantitative type of inheritance (Table 7) and 12 that follow a qualitative type of inheritance (Table 8). The characterization of entries according to the 24 phenotypic descriptors was in accordance with UPOV guideline TG/12/9 Rev. 2 of the species [63] for distinctness, uniformity and stability, and the descriptor list for common bean published by Bioversity International [64]. Observations/measurements were recorded at standardized phenological stages from ten randomly selected plants from each entry. The evaluated traits included vegetative-, reproductive- and yield-related parameters. The morphological traits were used to evaluate the intra- and inter-phenotypic variation and to identify discrete characters distinguishing commercial varieties from local landraces, given the need to conserve and utilize local plant resources in light of genetic base narrowing. On the other hand, yield components were recorded at full maturity to determine the yield potential of each entry and select superior genotypes.

In parallel, the seven common bean entries were screened phenotypically for resistance to BCMV at three developmental stages covering the entire biological cycle (V4, R6 and maturity). At each observation timepoint, every symptomatic expression was recorded in detail, including the nature, severity, and progression of symptoms, ensuring that plants which may have presented delayed or atypical symptom development were not classified as resistant. The disease resistance scoring was initiated immediately after the first symptom’s appearance in the field trials. Therefore, virus infection originated either through aphid transmission or through infected seeds. The total number of individual plants per entry was monitored using viral resistance scoring based on visual disease symptoms, to facilitate the subsequent selection process (Figure 3). Approximately 20 plants per entry—which tested positive for BCMV infection—were used as a sample size for the subsequent frequency distribution analysis. Symptoms such as mosaic, mottling, leaf deformation, vein banding and necrosis were rated utilizing a 1–3 observation scale (susceptibility index) as follows: 1—mosaic and/or leaf rolling; 2—hypersensitive necrotic reactions including top necrosis, vein necrosis and/or small necrotic lesions; 3—no symptoms. This simplified observation scale, widely adopted in related common bean studies [31], was used to classify genotypes as susceptible, resistant with mild symptoms and resistant without symptoms. Finally, the susceptibility index served as a baseline for validating molecular resistance diagnostics (Section 3.3).

The 20 plants per entry mentioned above were tested molecularly for the presence of BCMV by reverse-transcription polymerase chain reaction (RT-PCR) using primers [BCMV-CP-UP (5′-AAATGTGGTACAATGCTGTGAAGG-3′) and BCMV-CP-DO (5′-TCAG-TATTCTCGCTGGTTGTTGC-3′)] targeting a 468 bp region of the coat protein gene according to Chiquito-Almanza et al. (2017) [65]. For this purpose, total RNA was extracted from leaf material originating from the bean plants using the protocol of Ruiz-García et al. (2019) [66], as modified by Panailidou et al. (2023) [67]. PCR amplicons were analyzed on a 1.5% agarose gel previously stained with Midori Green Advance gel (Nippon, Dueren, Germany) and visualized under UV light. Additionally, the same samples were also tested for the presence of Alfalfa mosaic virus (AMV), Cucumber mosaic virus (CMV) and Bean yellow mosaic virus (BYMV); however, no positive plants were detected.

Accurate phenotyping in the field and effective selection for both plant yield potential and stability of performance in the same generation were accomplished by prognostic breeding [62] and pure-line selection. Yield potential and genotype stability were quantified in terms of seed weight per plant (g), using the Line prognostic equation (LPE) and Plant prognostic equation (PPE), according to the principles of the honeycomb selection design [68]. Biomass (g plant^−1^) and harvest index were also estimated. The Plant prognostic equation (PPE), as the product of the plant yield index (PI) and stability index (SI), was used for the selection of the best plants in each entry. On the other hand, the Line prognostic equation (LPE) was used for ranking the studied lines/entries and promoting only the best ones. In this concept, intrapopulation single-plant selection was carried out effectively by increasing selection pressure to only keep the superior genotypes.

### 3.3. The Molecular Approach—SSR Genetic Diversity and Molecular Characterization of Resistance to BCMV Based on CAPS and SCAR Markers

Genomic DNA was extracted from young trifoliate leaves of five representative plants per entry using the CTAB protocol [69], followed by spectrophotometric quality control. DNA integrity was verified by 1.0% agarose gel and finally the DNA samples were diluted to a concentration of 20 ng/μL.

Five highly polymorphic Simple Sequence Repeat (SSR) markers, previously re-ported for *P. vulgaris* diversity studies [70], were selected to provide strong discrimination power (Table 9). Each forward primer was labeled with fluorescent dye to simplify the determination of alleles per locus in a fragment analysis carried out in a 3500 Genetic Analyzer (Applied Biosystems, Waltham, MA, USA). PCR amplification reactions were performed in a SureCycler 8800 Agilent (Agilent Technologies, Santa Clara, CA, USA) thermocycler. The final reaction volume was 20 μL and contained the following reagents: 2 μL of 10× PCR Buffer solution, 0.4 μL DNTPs (10 mM), 0.5 μL of each primer (forward and reverse), 0.2 μL kapa Taq DNA polymerase (5 U/μL) and 1 μL DNA (20 ng). The PCR reactions were run as follows: 2 min at 95 °C for the initial denaturation, followed by 35 cycles consisting of 94 °C for 1 min, 47 °C or 48 °C or 49 °C for 1 min (depending on the primer pair), and 72 °C for 1 min, and a final extension step of 72 °C for 10 min. The amplified fragments were separated in 2.0% agarose gel, stained with ethidium bromide, and visualized under a UV light MiniBIS (DNR Bio-Imaging Systems Ltd., Jerusalem, Israel). Fragment analysis reactions were carried out in Applied Biosystems MicroAmp Optical 96-Well Reaction Plates containing 12.5 μL HiDi Formamide (Applied Biosystems, Thermo Fischer Scientific, Waltham, MA USA) as buffer, 0.5 μL 600 LIZTM Size Standard, and 2 μL 1:10 DNA for each sample.

Resistance to BCMV was assessed using Sequence-Characterized Amplified Region (SCAR) and Cleaved Amplified Polymorphic Sequence (CAPS) markers (Table 10) linked to major resistance loci, such as the dominant *I* locus and *bc-3* recessive gene, previously used in related studies [23,24,26]. The five plants per entry that were screened with molecular markers came from the group of 20 plants/entry that tested positive for BCMV and were phenotypically characterized under a honeycomb arrangement. The final reaction volume was 25 μL and contained the following reagents: 2 μL of 10× PCR Buffer solution, 0.4 μL DNTPs (10 mM), 0.5 μL of each primer (forward and reverse), 0.2 μL Taq DNA polymerase (5 U/μL) and 1 μL DNA (20 ng). The PCR thermal profile sequence contained a first step of 95 °C for 4 min and 35 cycles of 94 °C for 30 s, 55 °C or 56 °C or 59 °C for 30 s (depending on the primer pair) and 72 °C for 30 s, and a final extension step of 72 °C for 6 min. In the case of codominant markers, restriction enzymes TaqI and RsaI were used after PCR to identify individuals carrying resistant alleles for the *I* locus and *bc-3* gene, respectively. Each reaction contained 5 μL DNA/PCR product, 5 μL 10× NE Buffer (CutSmart), and 1 μL TaqI or RsaI enzyme, and the final volume was adjusted to 50 μL/sample. The samples were then incubated at 65 °C or 37 °C overnight, as this is the optimal reaction temperature in each case for the restrictive endonucleases TaqI and RsaI. The visualization of enzyme activity and the presence of a double band was confirmed in 2.0% agarose gel. Finally, Sanger sequencing was performed in an ABI3730xl DNA Analyzer (Applied Biosystems, Waltham, MA, USA).

### 3.4. Statistical Analysis and Visualization

Regarding the morphological diversity analysis, descriptive statistics and frequency distributions were calculated for the 12 quantitative traits. Normality of data was assessed using the Shapiro–Wilk test. For the 12 qualitative traits, frequency distributions were examined, while subsequent chi-square tests were performed to assess differences between genotypes. Analysis of variance (ANOVA) was performed for quantitative traits to test for significant differences between genotypes (F-test; *p* < 0.05) followed by the Skott–Knott ESD test (*p* < 0.01) for grouping entries into homogeneous clusters based on the mean performance for each trait. Pearson’s correlation coefficient was also calculated for all pairwise combinations of traits. Biological replicates were averaged per entry for multivariate analyses. Gower’s dissimilarity matrix was calculated to account for both quantitative and qualitative traits simultaneously. The optimal number of clusters based on Gower’s dissimilarity was estimated by Silhouette analysis.

For SSR markers (five loci; two alleles per locus), Nei’s genetic distance was computed after selecting a consensus allele per entry and molecular analysis of variance was performed. Molecular diversity indices, including the number of different alleles (Na), observed (Ho) and expected (He) heterozygosity, Shannon’s Information Index (SI) and Polymorphism Information Content (PIC) were calculated per locus. UPGMA clustering was performed on both morphological and molecular distance matrices, with dendrogram reliability assessed using cophenetic correlation coefficients. Principal Coordinate Analysis (PCoA) was conducted on both datasets. Comparative analysis between morphological and molecular diversity patterns included the Mantel test (9999 permutations) for the correlation of distance matrices and a Procrustes analysis (9999 permutations) to assess ordination congruence. Finally, an integrated analysis combined both distance matrices (50:50 weight after standardization) for UPGMA clustering and the cluster assignments were compared across the three approaches. The Genotypic Coefficient of variation was estimated as described by Burton et al. [51]. Broad-sense heritability (*H*^2^), along with standard errors, was computed using the formula given by Ward et al. [71]. Finally, genetic advance as a percentage of mean (GAM) was computed as described by Johnson et al. [54].

In terms of phenotypical estimation of resistance, the nominal resistance scores were converted into categorical factors for subsequent analyses. Frequency distributions and proportions were calculated for each resistance category to characterize the overall resistance profile of the tested germplasm. Entry-specific summaries were generated by computing the frequency and percentage of each resistance phenotype within entries, while the most frequent resistance response for each entry was determined. In this way, a consensus phenotype for each entry was provided, simplifying the classification into broad resistance categories (resistant/tolerant/susceptible). Chi-squared goodness-of-fit tests assessed whether the resistance categories were uniformly distributed across all observations. Association between entry/genotype and resistance phenotype was evaluated using chi-squared tests of independence on contingency tables. At the molecular level, a comparative analysis of the sequencing data was performed, which included alignment of the sequences for each marker.

Specifically, for the application of prognostic breeding and selection of superior genotypes, the crop yield potential of each individual plant was measured based on the following equation (Plant prognostic equation, PPE), using the Prognostic Breeding Application-Honey Design Selection add-in [62]:

PPE=(xx¯r)2·(x¯gs)2

In addition to the PPE, the Line prognostic equation (LPE) was used for the ranking of the entries and selection of the best lines:

LPE=(x¯gx¯t)2·(x¯gs)2

In the above formulas, x is the yield of each plant, ${\bar{x}}_{r}$ is the mean yield of the surrounding plants within the specified moving ring, ${\bar{x}}_{g}$ and *s* denote the mean plant yield and standard deviation, respectively, of the line’s plants laid out in a moving grid, and ${\bar{x}}_{t}$ is the mean plant yield of the whole trial.

All statistical analyses were performed in R software version 4.2.2. The following packages were used: agricolae, tidyverse, dplyr, readxl, writexl, cluster, adegenet, poppr, pegas, ape, hierfstat, vegan, ggplot2, gridExtra, corrplot, RColorBrewer, gridExtra, and scales.

For the SSR diversity analysis, Geneious Prime 2023.2 was used for the determination of allele calls, while GenAlEx 6.51b2 was used in addition to R packages. JMP18 with Prognostic Breeding Application-Honey Design Selection add-in [62] was utilized for honeycomb selection parameters.

## 4. Conclusions

The alternative strategy of resistance phenotypical screening in real-time field conditions in the absence of competition (honeycomb prognostic breeding methodology), along with systematic molecular diagnostics and MAS (SSR, SCAR, and CAPS), allowed for the discrimination of elite genotypes, and this study concluded with preliminary findings for the adoption of such a pipeline. However, future research directions include validation of the initial results through controlled environmental conditions with defined viral strains, to clarify the relative contribution of genetic and environmental factors to phenotypic variation. Also, long-term field evaluation in different locations is proposed to control the adaptability of superior lines. Our study highlights the considerable morpho-agronomic and resistance characteristics of local Greek common bean (*P. vulgaris* L.), demonstrating its position in the Mediterranean common bean germplasm context and emphasizing the need for conservation. The tested materials can serve multiple roles in breeding programs as promising parents for the development of resilient high-yielding common bean varieties, especially in the context of a climate crisis. The findings underscore both the practical value of early systematic phenotyping screening combined with MAS and the critical importance of conserving landrace diversity as a reservoir of valuable resistance and yield alleles for crop improvement and sustainability in agriculture.

## Figures and Tables

**Table 1:** Morphological and agronomic performance for the 12 quantitative traits, as defined by ANOVA genotype differences (*** *p* < 0.001; ** *p* < 0.01; * *p* < 0.05; a = 0.05) and Skott–Knott hierarchical mean clustering (*p* < 0.01) along with an estimation of genetic parameters. Means followed by the same letter in the column indicate groups of similar entries according to Skott–Knott. Standard errors (SEs) for heritability are presented.

No	Entry ID	PH		LL		LW		PW	PL		PT		PLB		NPP		NSP		SL		SW		W100S	
G1	Pyrgetos	121.0 ± 22.6	a	8.4 ± 1.5	b	6.9 ± 0.4	b	11.2 ± 1.9	101.0 ± 18.2	a	5.2 ± 1.3	b	7.0 ± 1.6	c	93.6 ± 38.6	a	3.8 ± 0.8	a	10.2 ± 1.1	c	6.8 ± 0.5	b	34.6 ± 10.9	b
G2	Florina	116.8 ± 19.2	a	8.0 ± 1.6	b	6.8 ± 1.2	b	9.8 ± 1.6	107.0 ± 9.8	a	5.2 ± 1.6	b	7.6 ± 1.8	c	49.2 ± 13.4	b	2.4 ± 0.9	b	9.8 ± 0.8	c	7.0 ± 0.7	b	57.3 ± 18.9	a
G3	Cannellino	52.8 ± 5.3	c	9.9 ± 0.7	a	8.9 ± 0.6	a	9.2 ± 1.9	103.4 ± 11.2	a	6.4 ± 0.6	a	14.0 ± 3.3	a	37.8 ± 14.1	b	2.2 ± 0.5	b	13.4 ± 1.1	a	6.6 ± 0.6	b	63.1 ± 11.2	a
G4	Lingot	48.5 ± 7.5	c	8.3 ± 1.0	b	6.6 ± 0.5	b	10.4 ± 1.7	79.0 ± 15.2	b	7.4 ± 1.1	a	15.0 ± 1.2	a	45.8 ± 18.5	b	2.2 ± 0.5	b	13.6 ± 1.1	a	6.6 ± 0.6	b	54.1 ± 6.4	a
G5	Kileler	101.0 ± 6.6	b	7.7 ± 0.7	b	6.1 ± 0.5	c	11.2 ± 1.8	101.0 ± 10.3	a	5.6 ± 0.6	b	9.0 ± 1.7	c	68.6 ± 22.9	b	3.8 ± 0.8	a	11.6 ± 0.6	b	8.2 ± 0.8	a	30.2 ± 6.2	b
G6	Karatzova	49.7 ± 8.2	c	6.8 ± 0.6	b	5.5 ± 0.4	c	10.6 ± 1.3	88.0 ± 10.4	b	6.4 ± 0.6	a	11.0 ± 1.4	b	61.6 ± 25.2	b	1.6 ± 0.6	b	7.8 ± 0.8	d	5.2 ± 0.5	c	47.8 ± 15.4	a
G7	Smyrni	93.4 ± 9.1	b	7.4 ± 0.7	b	5.9 ± 0.4	c	11.0 ± 1.7	95.0 ± 10.0	a	5.8 ± 0.8	b	8.2 ± 1.8	c	52.2 ± 21.3	b	2.4 ± 1.1	b	10.2 ± 0.5	c	8.2 ± 1.1	a	69.1 ± 29.3	a
	Mean	83.3 ± 32.4		8.1 ± 1.3		6.7 ± 1.1		10.5 ± 1.7	96.3 ± 14.6		6.0 ± 1.2		10.3 ± 3.5		58.4 ± 27.4		2.6 ± 1.1		10.9 ± 2.1		6.9 ± 1.2		50.9 ± 19.8	
	F-statistic	***		**		***		ns	*		*		***		*		***		***		***		**	
**Genetic parameters**																								
	GCV (%)	38.1		10.8		15.8		0.0	8.4		10.8		29.8		26.2		29.3		18.6		14.3		24.5	
	*H*^2^	0.9 ± 0.1		0.4 ± 0.4		0.7 ± 0.2		0.0 ± 0.6	0.3 ± 0.4		0.3 ± 0.5		0.7 ± 0.2		0.3 ± 0.4		0.5 ± 0.3		0.8 ± 0.1		0.7 ± 0.2		0.4 ± 0.4	
	GAM (%)	72.5		14.2		27.6		0.0	9.4		11.9		51.5		29.5		42.4		35.0		24.0		31.0	

PH—plant height (cm); LL—leaflet length (cm); LW—leaflet width (cm); PW—pod width (mm); PL—pod length (mm); PT—pod thickness (mm); PLB—pod length of beak (mm); NPP—number of pods per plant (un); NSP—number of seeds per pod (un); SL—seed length (mm); SW—seed width (mm); W100S—weight of 100 seeds (g).

**Table 2:** Details of SSR primers used, number of different alleles (Na), number of effective alleles (Ne), Shannon’s Information Index (I) and Polymorphism Information Content (PIC) in 7 common bean entries.

SSR Primer	Na	Ne	Shannon Index (I)	Ho	He	PIC
PhC-X04660	1.857	1.691	0.419	0.000	0.246	0.562
PhC-AZ044945	2.571	2.148	0.819	0.514	0.518	0.646
PhC-J01263	1.000	1.000	0.000	0.000	0.000	0.000
PhC-AY298744	3.143	2.388	0.891	0.271	0.495	0.737
PhC-AZ301561	1.000	1.000	0.000	0.000	0.000	0.000
**Mean**	**1.914**	**1.646**	**0.426**	**0.157**	**0.252**	**0.389**

**Table 3:** Entry-specific resistance profiles based on symptomatology recorded after phenotypically screening 20 plants per entry tested for BCMV infection. The presence/absence of resistance genes is also highlighted.

Entry	Scale 1—Severe Symptoms	Scale 2—Mild Symptoms	Scale 3—No Symptoms	Dominant *I* Gene	Recessive *bc-3* Gene
G1	25%	30%	45%	Yes	Yes
G2	35%	55%	10%	No	No
G3	30%	55%	15%	Yes	No
G4	50%	40%	10%	No	No
G5	15%	50%	35%	Yes	No
G6	35%	65%	-	Yes	No
G7	5%	70%	25%	No	No

**Table 4:** Potential allele combinations based on amplification products, phenotypical disease rating and representative symptomatology recorded for 5 samples/entry that were screened with CAPS and SCAR markers.

Plant/Sample ID	Allele Combination	Disease Rating	Phenotype (Symptomatology)
G1-1	ii	susceptible	severe symptoms: mosaic and leaf rolling
G1-2	ii & bc-3	resistant	no symptoms
G1-3	ii	susceptible	severe symptoms: mosaic
G1-4	ii	susceptible	severe symptoms: leaf rolling
G1-5	II	resistant	no symptoms
G2-1	ii	resistant	no symptoms
G2-2	ii	tolerant	mild symptoms: vein necrosis
G2-3	ii	tolerant	mild symptoms: vein necrosis
G2-4	ii	tolerant	mild symptoms: small necrotic lesions
G2-5	ii	tolerant	mild symptoms: vein necrosis
G3-1	ii	tolerant	mild symptoms: small necrotic lesions
G3-2	ii	tolerant	mild symptoms: top necrosis
G3-3	ii	susceptible	severe symptoms: mosaic
G3-4	II	resistant	no symptoms
G3-5	ii	susceptible	severe symptoms: mosaic
G4-1	ii	susceptible	severe symptoms: mosaic
G4-2	ii	susceptible	severe symptoms: systemic necrosis
G4-3	ii	susceptible	severe symptoms: systemic necrosis
G4-4	ii	susceptible	severe symptoms: mosaic
G4-5	ii	tolerant	mild symptoms: top necrosis
G5-1	II	resistant	no symptoms
G5-2	II	resistant	no symptoms
G5-3	Ii	tolerant	mild symptoms: mild mosaic
G5-4	Ii	tolerant	mild symptoms: vein necrosis
G5-5	Ii	tolerant	mild symptoms: vein necrosis
G6-1	Ii	tolerant	mild symptoms: mild mosaic
G6-2	Ii	tolerant	mild symptoms: mild mosaic
G6-3	Ii	tolerant	mild symptoms: vein necrosis
G6-4	ii	susceptible	severe symptoms: mosaic and leaf rolling
G6-5	Ii	tolerant	mild symptoms: small necrotic lesions
G7-1	ii	resistant	no symptoms
G7-2	ii	resistant	no symptoms
G7-3	ii	tolerant	mild symptoms: mild mosaic
G7-4	ii	tolerant	mild symptoms: top necrosis
G7-5	ii	tolerant	mild symptoms: small necrotic lesions

**Table 5:** Ranking of evaluated entries utilizing the prognostic breeding parameters (SI—Stability Index; LI—Line Yield Index; LPE—Line Prognostic Equation; PI—Plant Yield Index; PPE—Plant Prognostic Equation).

Code	Entry	Mean	Mean %	Std Dev	SI	SI%	LI	LI%	LPE	LPE%	Mean PI	Mean % PI	Mean PPE	Mean % PPE
G1	Pyrgetos	65.0	100 ^1^	38	2.9	94.4	2.35	100	6.88	100	3.3	100	9.3	100
G2	Florina	41.5	63.9	23.6	3.1	100	0.96	40.8	2.98	43.2	1.4	43.8	4.3	46.4
G7	Smyrni	42.6	65.5	25.5	2.8	89.7	1.01	42.9	2.80	40.7	1.4	45.3	4.0	43.0
G6	Karatzova	42.3	65.0	31.4	1.8	58.5	0.99	42.3	1.80	26.2	1.9	60.3	3.5	37.4
G5	Kileler	37.0	56.9	32.0	1.3	43	0.76	32.4	1.02	14.8	1.2	37.9	1.6	17.3
G3	Cannellino	29.4	45.3	18.9	2.4	78.3	0.48	20.5	1.17	17.0	0.6	18.3	1.4	15.2
G4	Lingot	29.0	44.7	19.5	2.2	71.8	0.47	20	1.04	15.2	0.5	15	1.1	11.4

^1^ Values are expressed as percentages based on the entry with the highest value.

**Table 6:** The seven common bean entries evaluated in this study.

Entry	Common Name	Type	Origin
G1	Pyrgetos	Old commercial variety	Greece
G2	Florina	Landrace	Greece
G3	Cannellino	Commercial variety	Italy
G4	Lingot	Commercial variety	France
G5	Kileler	Population	Greece
G6	Karatzova	Landrace	Greece
G7	Smyrni	Landrace	Greece

**Table 7:** Quantitative descriptors used in the characterization of the seven entries of *Phaseolus vulgaris* L.

Descriptor	Method of Evaluation
plant height—PH (cm)	Measured from cotyledon scar to the tip of the plant at maturity.
leaflet length—LL (cm)	Measured on the terminal leaflet of the third trifoliate leaf from pulvinus to leaf tip.
leaflet width—LW (cm)	Measured on the terminal leaflet of the third trifoliate leaf from one edge to the other.
pod width—PW (mm)	Measured from the largest fully expanded immature pods.
pod length—PL (mm)	Measured from the largest fully expanded immature pods excluding beak.
pod thickness—PT (mm)	Measured from the largest fully expanded immature pods.
pod length of beak—PLB (mm)	Measured from the end of the last loculus.
number of pods/plant—NPP	Counted after flowering, between 40 and 50 days, with well-developed pods.
number of seeds/pod—NSP	Counted after harvest with the pod completely dry.
seed length—SL (mm)	Measured parallel to the hilum.
seed width—SW (mm)	Measured perpendicular to the hilum.
weight of 100 seeds—W100S (g)	Measured after harvest, to the nearest first decimal place, with a moisture content of 12–14%.

**Table 8:** Qualitative descriptors used in the characterization of the seven entries of *Phaseolus vulgaris* L.

Title 1	Title 2
Plant: anthocyanin coloration of hypocotyl	1 = absent; 9 = present
Dwarf beans: plant type	1 = non-trailing; 2 = trailing
Terminal leaflet: shape	1 = triangular; 2 = triangular to circular; 3 = circular; 4 = circular to rhombic; 5 = rhombic
Flower: color of standard	1 = white; 2 = pinkish white; 3 = pink; 4 = violet
Flower: color of wing	1 = white; 2 = pinkish white; 3 = pink; 4 = violet
Pod: shape in cross section (through seed)	1 = elliptic; 2 = ovate; 3 = cordate; 4 = circular; 5 = eight-shaped
Pod: ground color	1 = yellow; 2 = green; 3 = violet
Pod: stringiness of ventral suture	1 = absent; 9 = present
Pod: shape of distal part (excluding beak)	1 = acute; 2 = acute to truncate; 3 = truncate
Seed: shape in longitudinal section	1 = circular; 2 = circular to elliptic; 3 = elliptic; 4 = kidney-shaped; 5 = rectangular
Seed: number of colors	1 = one; 2 = two; 3 = more than two
Seed: main color (largest area)	1 = white; 2 = green or greenish; 3 = gray; 4 = yellow; 5 = beige; 6 = brown; 7 = red; 8 = violet; 9 = black

**Table 9:** List of 5 SSR markers used in molecular analysis to assess genetic diversity among the 7 common bean entries evaluated in this study.

Primer Code	Sequence	Annealing Temperature (°C)	References
PhC-X04660a-F	TTGATGACGTGGATGCATTGC	48	[70]
PhC-X04660a-R	AAAGGGCTAGGGAGAGTAAGTTGG		
PhC-J01263a-F	ATGCATGTTCCAACCACCTTCTC	49	[70]
PhC-J01263a-R	GGAGTGGAACCCTTGCTCTCTCATC		
PhC-AZ044945b-F	CATCAACAAGGACAGCCTCA	47	[70]
PhC-AZ044945b-R	GCAGCTGGCGGGTAAAACAG		
PhC-AZ301561b-F	CAGTAAATATTGGCGTGGATGA	47	[70]
PhC-AZ301561b-R	A TGAAAGTGCAGAGTGGTGGA		
PhC-AY298744b-F	CATAACATCGAAGCCTCACAGT	47	[70]
PhC-AY298744b-R	ACGTGCGTACGAATACTCAGTC		

**Table 10:** List of 3 resistance-associated markers used in the present study (dominant SCAR SW13 marker for the *I* gene; codominant BCMV-CAPS marker for the *I* gene; codominant marker for the *bc-3* gene (eucaryotic translation initiation factor eIF4E)).

Primer Code	Sequence	Annealing Temperature (°C)	References
BCMV_CAPS _F	5′-AGGAGGAAGAACGGTGGTC-3′	56	[24]
BCMV_CAPS _R	5′-TTTGGTGGTAATTTGAAAATGG-3′		
SW13_F	5′-CACAGCGACATTAATTTTCCTTTC-3′	59	[23]
SW13_R	5′-CACAGCGACAGGAGGAGCTTATTA-3′		
ENM-FWe	5′-ACCGATGAGCAAAACCCTA-3′	55	[26]
ENM-Rve	5′-CAACCAACTGGTATCGGATT-3′		

## Data Availability

The original contributions presented in this study are included within the article. The underlying datasets supporting the findings are available from the corresponding authors upon reasonable request.

## Abbreviations

The following abbreviations are used in this manuscript:

BCMV	Bean common mosaic virus
MAS	Marker-assisted selection
SSR	Simple sequence repeat
CAPS	Cleaved amplified polymorphic sequence
SCAR	Sequence-characterized amplified region
GCV	Genetic coefficient of variation
WPN	Whole-plant necrosis
BSA	Bulked segregant analysis
CIAT	International Center for Tropical Agriculture
ANOVA	Analysis of variance
PH	Plant height
LL	Leaflet length
LW	Leaflet width
PW	Pod width
PL	Pod length
PT	Pod thickness
PLB	Pod length of beak
NPP	Number of pods per plant
NSP	Number of seeds per pod
SL	Seed length
SW	Seed width
W100S	Weight of 100 seeds
*H*^2^	Broad-sense heritability
GAM	Genetic advance as a percentage of mean
UPGMA	Unweighted pair group method with arithmetic mean
CCC	Cophenetic correlation coefficient
DNA	Deoxyribonucleic acid
PCoA	Principal coordinate analysis
Na	Number of different alleles
Ne	Number of effective alleles
I	Shannon’s information index
PIC	Polymorphism information content
HR	Hypersensitive response
QTL	Quantitative trait locus
SI	Stability index
LI	Line yield index
LPE	Line prognostic equation
PPE	Plant prognostic equation
PI	Plant yield index
RT-PCR	Reverse-transcription polymerase chain reaction
AMV	Alfalfa mosaic virus
CMV	Cucumber mosaic Virus
BYMV	Bean yellow mosaic virus
SNP	Single-nucleotide polymorphism
UPOV	International Union for the Protection of New Varieties of Plants
CTAB	Cetyltrimethylammonium bromide
